# Malignant Dysrhythmias – Brugada Type 1 Pattern Formation in the Presence of Fever

**DOI:** 10.51894/001c.5072

**Published:** 2016-10-24

**Authors:** Joshua Liroff, Karyn Gilbert, Brian S. Kim

**Affiliations:** 1 Emergency Department, Henry Ford Allegiance Health, Jackson, MI

**Keywords:** case study, febrile states, fevers, cardiac arrhythmia, brugada syndrome, brugada

## Abstract

Brugada is a malignant cardiac dysrhythmia characterized by ST elevations in precordial leads V1 to V3. It is an autosomal dominant disease, and patients are usually asymptomatic. Often the initial presenting symptom of this syndrome is syncope or sudden cardiac death (SCD). In patients with this syndrome, the only definitive treatment is the implantation of a cardioverter-defibrillator device (ICD). Previously it has been reported that Brugada type EKG patterns have been observed in patients who have used sodium channel blocking medications/drugs and in patients who are febrile. In this case, a 58 year-old male presented to our emergency department with a fever of five days duration and a non-productive cough. The patient was initially diagnosed with bilateral pneumonia, however during the initial workup his EKG demonstrated a Brugada type EKG pattern. The patient did not have any history of cardiac disease, nor was there any history of syncope or SCD in his family. The patient was eventually discharged from the hospital four days after his initial presentation and instructed to follow up with cardiology. Brugada type EKG patterns are known to occur in patients in febrile states and in those patients using certain types of medications. In those patients who present under the above mentioned conditions who are otherwise asymptomatic, the literature does not support the implantation of an ICD.

## INTRODUCTION

Brugada is a cardiac syndrome characterized by sudden cardiac death, usually in young healthy adults by ventricular dysrhythmias. It is an autosomal dominant disease with mutations in the SCN5A and SCN10A subunits of the SCN gene that encode for subunits of cardiac sodium channels. Typical EKG findings in Brugada Syndrome usually demonstrate a “pseudo right bundle branch block” or “shark fin-like” appearance with ST elevations in precordial leads V1 to V3.^1–5^ Patients with Brugada often are asymptomatic. The syndrome usually manifests itself between the ages of 22-65, and often presents with either syncope or sudden cardiac death (SCD).^2^ Early recognition of this dysrhythmia is critical in the prevention of SCD,^6^ as patients can be started on antiarrhythmic therapy or undergo the implantation of a cardioverter-defibrillator device (ICD). Previously, Brugada type EKG patterns have been observed in patients during febrile states and in those patients who are under the influence of sodium channel blocking agents such as cocaine, class I antiarrhythmics, anesthetics, and tricyclic antidepressants.^7–12^

In our case study, we present the case of a 58 year old male who initially presented to our emergency department with a fever of five days duration, a maximum temperature of 39.5°C, and a non-productive cough. This case further demonstrates the utility of electrocardiograms in the recognition of possible dysrhythmias in the febrile state.

## CASE

The patient was a 58 year-old male with a history of hypertension, hyperlipidemia and diabetes, who initially presented to our emergency department complaining of a fever, diarrhea and fatigue. According to the patient, these symptoms had been ongoing for the past 5 days. The patient also had a non-productive cough. The patient denied any chest pain, heart palpitations, shortness of breath, syncope, dizziness, or abdominal pain. The patient denied any previous syncopal episodes or any previous cardiac history including dysrhythmias, previous myocardial infarctions, coronary artery disease, or congestive heart failure. The patient denied any family history of early cardiac deaths or family history of heart disease. Review of the patient’s current medications at the time included atorvastatin, lisinopril-hydrochlorothiazide, and metformin.

The patient was febrile on presentation with a temperature of 39.5° C. He was tachycardic with a heart rate of 110. Patient was hemodynamically stable with a blood pressure of 108/67mmHg. Crackles were heard at the bases of his lungs bilaterally. The remaining physical exam was unremarkable.

**Figure 1:**
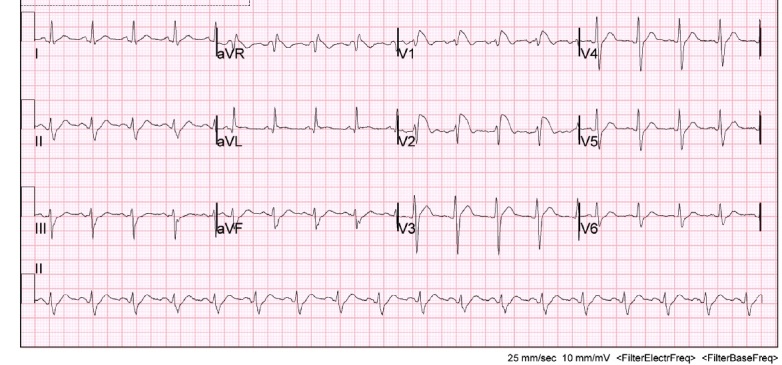
Patient’s initial EKG upon presentation to the emergency department showing a type 1 Brugada Pattern. Notice the coved segment ST elevations in V1 and V2.

**Figure 2:**
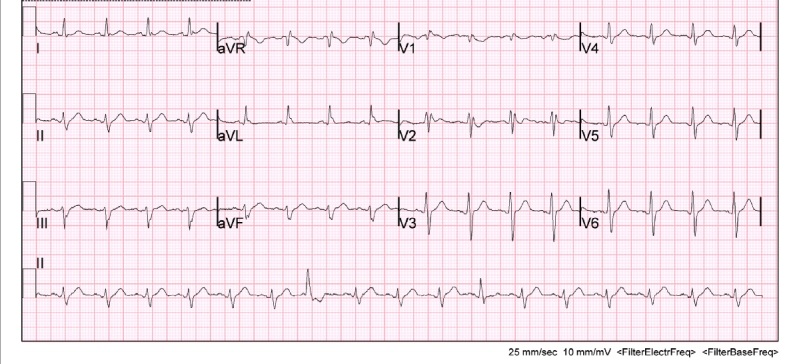
Patient’s previous EKG obtained from previous visit to emergency department two years earlier.

Initially there was concern for sepsis, given his history of fever for five days and diarrhea. An infectious/metabolic workup was performed, as our initial differential diagnosis included pulmonary disease (atelectasis, bronchitis, pneumonia, tuberculosis, empyema, pulmonary embolism), urinary tract infection, bacteremia, gastroenteritis, and viral syndrome. Also, as the patient was complaining of fatigue and had cardiac risk factors, it was important to expand our differential to rule out cardiac causes such as myocardial infarction, myocarditis, and life-threatening dysrthymias. A complete blood count, electrolyte panel, troponin, EKG, chest x-ray, and blood cultures were obtained. Of note, the patient had an elevated lactic acid of 3.8 mmol/l, a sodium of 128 mmol/l and a white blood cell count of 29.2 k/mm^3^. The remaining laboratory studies were unremarkable and within normal physiologic limits, including his potassium, calcium and troponin. Surprisingly, the patient’s EKG demonstrated a type 1 Brugada pattern (Figure 1). This was a new finding, as his previous EKG had shown an incomplete right bundle branch block (Figure 2). Cardiology was consulted and it was recommended an echocardiogram and a computed tomography (CT) angiogram of the chest be obtained in order to rule out a right ventricular infarct and pulmonary embolism, respectively. Echocardiogram showed a left ventricular ejection fraction of 70% with trace mitral and tricuspid regurgitation. There was no evidence of valvular pathology, hypokinesis, or thrombus. CT angiogram of the chest demonstrated a large opacity in the left lower lobe, and infiltrates in the right upper, middle and lower lobes consistent with pneumonia. There was no evidence of pulmonary embolism. The patient was started on ceftriaxone and azithromycin and admitted to the hospital for further evaluation and treatment for community-acquired pneumonia. The patient was discharged from the hospital four days later. He was started on aspirin and metoprolol per recommendations by cardiology and was instructed to follow up with them in their office two weeks later.

## DISCUSSION

This particular case of fever induced Brugada is of significant importance, as it helps emphasize the utility of an EKG as a screening tool to quickly identify possible ST elevated myocardial infarctions or life-threatening dysrhythmias. In this case, although the patient’s differential diagnosis was expansive, his symptoms were of infectious etiology. Obtaining an EKG in his initial workup quickly identified a possible life-threating dysrhythmia, which helped the clinician direct appropriate resources to this patient’s care.

As mentioned, Brugada is described as a pseudo right bundle branch block with persistent elevation in leads V1-V3. There are three known Brugada-like EKG patterns. In this specific case, the patient presented with a Brugada type-1 pattern, which is characterized by a coved ST segment elevation of at least 2 mm followed by an inverted T-wave in the right precordial leads.^1^ Fever has shown to be a causative factor in the development of a Brugada-like EKG pattern.^13^ As in the case described above, it is believed the patient’s illness contributed to the development of the type 1 Brugada pattern that was observed on his initial EKG upon presentation to the emergency department.

Brugada pattern and Brugada syndrome are distinguished by the presence or absence of symptoms. Those patients without symptoms are said to exhibit a Brugada-like pattern, while those with symptoms and meeting established clinical criteria are considered to have Brugada syndrome. Diagnostic criteria include the presence of a coved ST elevated segment of at least 2 mm in more than one precordial lead plus at least one of the following clinical criteria: documented ventricular fibrillation, polymorphic ventricular tachycardia, family history of sudden cardiac death at less than 45 years of age, family history of type 1 Brugada pattern EKG changes, inducible ventricular tachycardia during electrophysiology studies, unexplained syncope suggesting a tachyarrhythmia, or nocturnal agonal respirations.^14^

In those patients with Brugada syndrome, it is recommended that the patient be referred to an electrophysiologist for ICD implantation, which is effective in terminating life threatening ventricular dysrhythmias. Pharmacologic treatment has not been proven thus far to prevent SCD in Brugada syndrome, although there are some studies that show quinidine may be of some benefit.^15^ ICD implantation is the only definitive treatment. For those patients with a Brugada-like EKG pattern who are otherwise asymptomatic and do not meet diagnostic criteria, treatment with ICD is not recommended as there is no data to suggest ICD use in these patients.^15^ In this particular patient, only the presence of EKG changes, that of a type I Brugada pattern were observed. The patient was asymptomatic and did not meet clinical criteria for the diagnosis of Brugada syndrome. As such, based on current guidelines no treatment was recommended, only cardiology follow-up.

## WHY IS THIS CASE RELEVANT?

Brugada syndrome is recognized as a cause of SCD. It is a cardiac dysrhythmia that can deteriorate into a fatal ventricular arrhythmia.^16^ Understanding the conditions that can potentiate Brugada syndrome is important for the practicing clinician, but also for the patient.^16^ Triggers such as fever, intoxication (alcohol, cocaine, or cannabis), vagal stimulation, electrolyte imbalance, anesthetics (amitriptyline, lithium) and sodium channel blockers are known to potentiate this rhythm. Approximately 25% of all cases of Brugada syndrome are caused by mutations in the cardiac sodium channel gene SCN5A. The mutated sodium channels result in temperature-dependent ionic changes that cause characteristic Brugada EKG patterns during fever.^17^

## CONCLUSION

Brugada induced EKG changes have been observed in patients with a fever and in those patients using certain drugs/medications. In those patients with Brugada-like EKG patterns who are otherwise asymptomatic, no further treatment such as ICD implantation or antiarrhythmic medication use is recommended as there is no data available at this time to suggest benefit to these patients.

### Conflict of Interest

The authors declare no conflict of interest.
